# Breakthrough COVID-19 Infection Among the General Community, Frontline Workers, and Healthcare Workers During the Second and Third Wave in North India: A Longitudinal Study

**DOI:** 10.7759/cureus.58860

**Published:** 2024-04-23

**Authors:** Anurag Chaudhary, Priya Bansal, Mahesh Satija, Surinder Pal Singh, Vikram Kumar Gupta, Pranjl Sharma, Sarit Sharma, Sangeeta Girdhar

**Affiliations:** 1 Department of Community Medicine, Dayanand Medical College & Hospital, Ludhiana, IND

**Keywords:** covid-19, healthcare workers, frontline workers, adverse effects, breakthrough infection, covid-19 vaccination

## Abstract

Background: Vaccination is among the most important public health tools for preventing the harm caused by communicable diseases. This was particularly true in the case of COVID-19 vaccination during the COVID-19 pandemic. However, no vaccine is 100% effective, and all carry the risk of breakthrough infection in vaccinated individuals.

Methodology: This longitudinal observational study was done on COVID-19-vaccinated individuals at a vaccination site in a tertiary care hospital. The study participants were categorized into the general community, frontline workers, and healthcare workers and were followed up during the study period from June 2021 to May 2022 post-vaccination. They were interviewed by telephone regarding adverse effects and breakthrough infections post-vaccination during the second and third wave of the COVID-19 pandemic in India. Incidence of breakthrough infection was calculated in all three categories after they received their first, second, and booster doses of vaccination.

Results: Fever was the most common adverse effect among all the categories of participants after the first and second doses. Incidence of breakthrough infection after the second dose of vaccination among frontline workers (RR: 5.7, 95% CI: 0.7-44.2) and healthcare workers (RR: 18.9, 95% CI: 2.6-138.6) was observed to be higher compared to the general community, but no such difference was observed among the three categories after the first dose of vaccination.

Conclusions: The incidence of breakthrough infection was found to be the highest in healthcare workers, followed by frontline workers compared to the general community, justifying their work profile and the risk associated with it.

## Introduction

COVID-19 has affected hundreds of millions of people globally since it was declared a pandemic by the World Health Organization on March 11, 2020 [1]. The COVID‐19 pandemic has brought drastic changes in India, not only exposing the fragility of the already overburdened and under‐resourced health system but also highlighting the strengths of the healthcare system in the prevention of COVID-19 infection from vaccine development and vaccine delivery through the successful vaccination program [2].

Vaccination is among the most important public health tools for reducing the spread and harm caused by dangerous diseases [3]. During the COVID-19 pandemic, several COVID-19 vaccines were validated and given emergency approval by the WHO. Before approval, a vaccine is assessed to ensure that it meets acceptable standards of quality, safety, and efficacy using clinical trial data, manufacturing data, and quality control processes. The assessment weighs the threat posed by the emergency and the benefit accrued against any potential risks. In line with their national regulations and legislation, countries have the autonomy to issue emergency use authorizations for any health product. Regarding COVID-19 vaccine usage in India, national regulatory authorities granted full or emergency use authorization for two COVID-19 vaccines [4] at the beginning of 2021.

On January 16, 2021, India launched the "World's Largest Vaccination Drive" with two vaccine candidates: COVISHIELD, the Indian version of the Oxford AstraZeneca vaccine, produced by the Serum Institute of India, and COVAXIN, India's homegrown inactivated COVID‐19 vaccine [5]. This program covered the entire length and breadth of the country and achieved the historic milestone of administering 200 crore COVID-19 vaccination doses as of 17 July 2022 [6].

The WHO has estimated that vaccines prevented at least 10 million deaths worldwide between 2010 and 2015 [7]. Although vaccines stop most people from getting seriously ill, no vaccine is 100% effective. Some people who have been vaccinated may experience "breakthrough infection," where they still get infected with the virus despite being fully vaccinated [8]. However, the risk of getting a breakthrough infection varies according to exposure to the infection, which happens to be maximum in healthcare workers. It is well known now that vulnerability to COVID-19 infection is highest among healthcare workers compared to other sections of the population [9].

Despite the availability of a vaccine and extensive vaccination, breakthrough infections were commonly noted in the vaccine-based protection against COVID-19. The present study was planned to study the incidence of COVID-19 infection post-vaccination among frontline workers, healthcare workers, and the general community.

## Materials and methods

**Figure 1:**
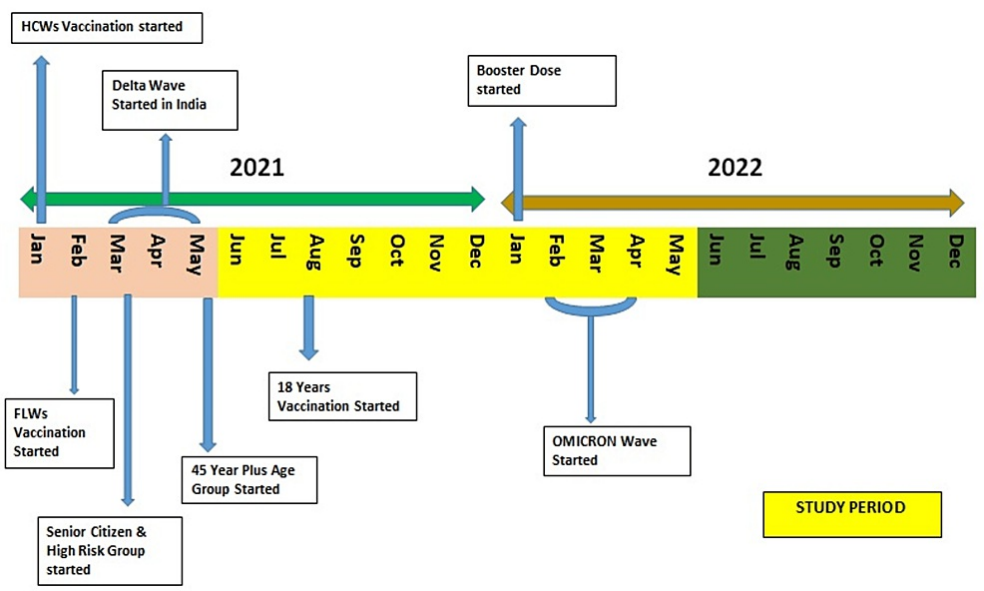
Timeline of COVID-19 vaccination with respect to Delta and Omicron waves FLW: frontline workers; HCW: healthcare workers.

The vaccination drive started in India in a phased manner with COVISHIELD [5], the Indian version of the Oxford AstraZeneca vaccine (Figure 1).

In our tertiary care hospital, vaccination was started on 16 January 2021 in collaboration with the district health authorities. Initially, it started with one session site and slowly the number of sites increased to four. Outreach vaccination sessions were also conducted by the teams as required by district health authorities. More than 2 lakh vaccinations were done at our vaccination center for COVID-19 till July 2022.

**Figure 2:**
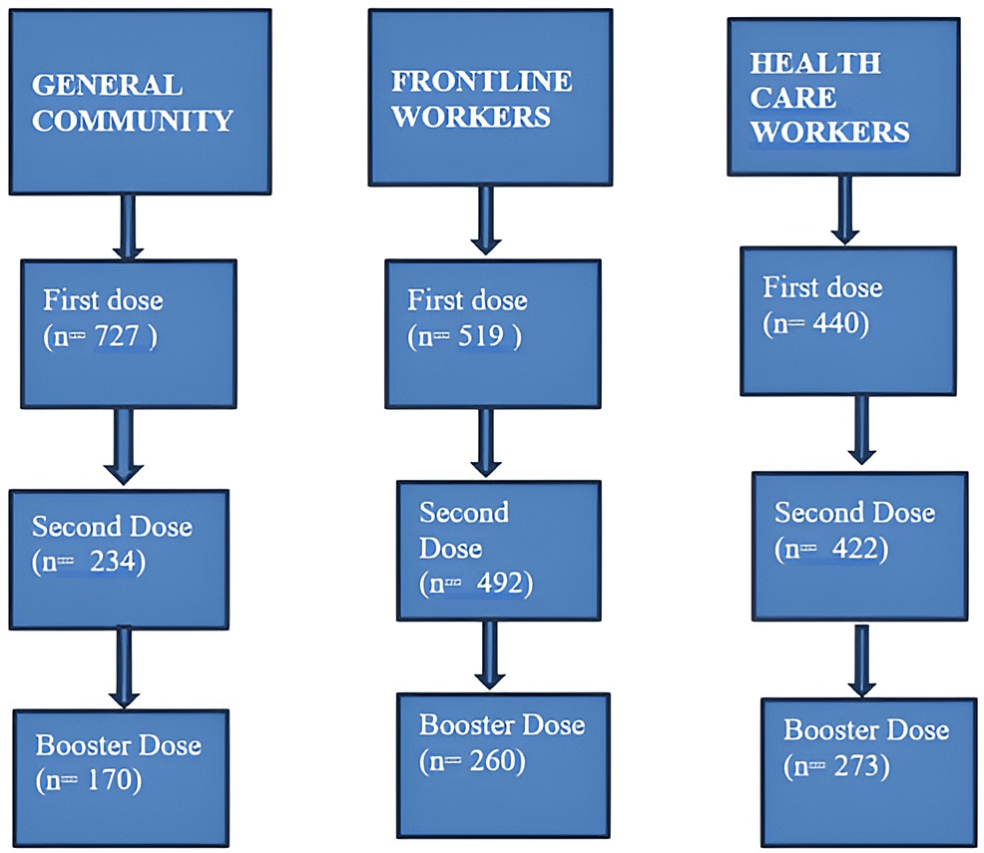
Study flowchart

The present longitudinal observation study was done on healthcare workers, frontline workers, and the general community as study participants from June 2021 to May 2022. A list of all those who got vaccinated with COVISHIELD vaccine till 31 May 2021 was prepared and segregated according to the following three categories: healthcare workers: healthcare providers and workers in healthcare settings (public and private, including Integrated Child Development Services (ICDS) workers) [10]; frontline workers: personnel from state and central police departments, armed forces, home guards, prison staff, disaster management volunteers, civil defense organizations, municipal workers, and revenue officials engaged in COVID-19 containment, surveillance, and associated activities [10]; and the general community. Because it was not possible to contact all those on the list by telephone, 10% of the subjects from each category were selected by systematic random sampling. The first subject was chosen by the currency note method and the further subjects were picked up per the sampling interval from the lists prepared for the three categories. The telephone numbers of all vaccine recipients were available to the authors because these were noted down before vaccination. The selected subjects were contacted by telephone and, in case of no response, the next subject on the list was contacted after two more unsuccessful attempts. A total of 4340, 5200, and 7249 persons were vaccinated under the three categories, i.e., healthcare workers, frontline workers, and the general community, respectively. The number of study subjects selected and interviewed was 440 healthcare workers, 525 frontline workers, and 727 general community, respectively, in the three categories. All selected study subjects were informed about the purpose of the telephone interview, and verbal consent was taken before asking questions (Figure 2).

The data were collected through a predesigned questionnaire with close-ended questions. Information regarding age profile, work profile, adverse effects after the first, second, and third dose, and breakthrough infection was requested. Upon interviewing the study participants, information about the adverse effects of COVID-19 and about positive status post-vaccination was taken. All those who refused to participate and those who did not pick up the phone when called thrice were excluded from the study. Later, follow-up was done during the study period post-vaccination, including a booster dose during the third wave (Omicron).

Due approval was received from the Institutional Ethics Committee (IEC), Dayanand Medical College & Hospital, Ludhiana, wide IEC No. 2021-662. The data were analyzed using SPSS (IBM Corp., Armonk, NY) [11]. Descriptive strategies were presented in percentages and mean ± standard deviations. A chi-square test was used to determine the difference between the categorical variables. Relative risk (RR), 95% confidence interval (95% CI), and p-values (p) were calculated to find the strength of association among the groups. All tests were two-tailed, and p < 0.05 was considered to be statistically significant.

## Results

Among the general community, more than half of the subjects (54.7%) were in the age group of 46-60 years, whereas among the frontline participants, most subjects were in the age group of 18-60 years. Among the healthcare participants, most (87.6%) were in the age group of 18-45 years. The mean ages of the general community, frontline workers, and healthcare workers were 58.87, 41.75, and 33.65 years, respectively. Among all the study participants, the proportion of males was more than females (Table 1).

**Table 1:** Demographic profiles of vaccinated healthcare workers, frontline workers, and the general community

Characteristics	General community (n = 727), N (%)	Frontline workers (n = 519), N (%)	Healthcare workers (n = 440), N (%)
Age			
18–45	45 (6.2)	261 (50.3)	385 (87.5)
46–60	398 (54.7)	255 (49.2)	55 (12.5)
>60	284 (39.1)	3 (0.6)	0 (0)
Mean ± SD	58.87 ± 18.9	41.75 ± 10.6	33.65 ± 8.7
Gender			
Male	382 (52.5)	349 (67.3)	294 (66.8)
Female	345 (47.5)	170 (32.7)	146 (33.2)

Table 2 depicts the adverse effects of COVID-19 vaccination after the first and second doses among the study participants. Among all the categories of participants, fever was a commonly presenting symptom after the first and second doses, and it was significantly higher after receiving the second dose as compared to the first dose. Symptoms like sore throat, aches, and pains were reported by all study participants, particularly after the first dose compared to the second dose.

**Table 2:** Adverse effects of first and second doses as reported by frontline workers, healthcare workers, and the general community

Side effects		General community, N (%)		Chi-square value (p-value)	Frontline workers, N (%)		Chi-square value (p-value)	Healthcare workers N (%)		Chi-square value (p-value)
		Adverse effects after the first dose	Adverse effects after the second dose		Adverse effects after the first dose	Adverse effects after the second dose		Adverse effects after the first dose	Adverse effects after the second dose	
1	Fever	532 (73.2)	192 (82.1)	7.5 (0.006)	420 (80.9)	443 (90.0)	16.8 (0.000)	346 (78.6)	390 (92.4)	32.8 (0.000)
2	Cough	34 (4.7)	13 (5.6)	0.3 (0.587)	23 (4.4)	11 (2.2)	3.7 (0.052)	8 (1.8)	4 (0.9)	1.2 (0.276)
3	Cough with expectoration	8 (1.1)	3 (1.3)	0.05 (0.820)	4 (0.8)	2 (0.4)	0.6 (0.453)	6 (1.4)	3 (0.7)	0.9 (0.345)
4	Tiredness	10 (1.4)	8 (3.4)	4.0 (0.044)	20 (3.9)	12 (2.4)	1.6 (0.199)	11 (2.5)	10 (2.4)	0.01 (0.901)
5	Aches and pains	23 (3.2)	2 (0.9)	3.7 (0.053)	10 (1.9)	5 (1.0)	1.4 (0.231)	11 (2.5)	2 (0.5)	5.9 (0.014)
6	Sore throat	129 (17.7)	16 (6.8)	16.4 (0.000)	82 (15.8)	20 (4.1)	38.3 (0.000)	24 (5.5)	15 (3.6)	1.8 (0.179)
7	Diarrhea	5 (0.7)	1 (0.4)	0.2 (0.660)	4 (0.8)	1 (0.2)	1.6 (0.198)	6 (1.4)	2 (0.5)	1.8 (0.173)
8	Conjunctivitis	7 (1.0)	2 (0.9)	0.02 (0.881)	1 (0.2)	0 (0.0)	-	4 (0.9)	0 (0.0)	-
9	Headache	9 (1.2)	1 (0.4)	-	10 (1.9)	2 (0.4)	4.9 (0.025)	7 (1.6)	3 (0.7)	1.4 (0.228)

The incidence of breakthrough infection after the first dose among the general community, frontline workers, and healthcare workers was 8.3, 7.7, and 9.1 per thousand participants, respectively. However, this difference was not statistically significant. The incidence of breakthrough infection after the second dose among the general community, frontline workers, and healthcare workers was 4.3, 24.4, and 80.6 per thousand participants, respectively. Frontline workers (RR: 5.7, 95% CI: 0.7-44.2) and healthcare workers (RR: 18.9, 95% CI: 2.6-138.6) had a higher risk of breakthrough infection after the second dose as compared to the general community. Similarly, incidence among healthcare workers was significantly higher after booster dose as compared to the general community (RR: 21.2, 95% CI: 2.9-156.1; Table 3).

**Table 3:** Risk of breakthrough infection amongst three categories of vaccinated study participants

Variables	Risk of breakthrough infection after the first dose			Risk of breakthrough infection after the second dose			Risk of breakthrough infection after the booster dose		
	General community	Frontline workers	Healthcare workers	General community	Frontline workers	Healthcare workers	General community	Frontline workers	Healthcare workers
No. of beneficiaries	727	519	440	234	492	422	170	260	273
Contracted COVID-19	6	4	4	1	12	34	1	2	34
Incidence per 1000	8.3	7.7	9.1	4.3	24.4	80.6	5.9	7.7	124.5
Relative risk	1	0.9	1.1	1	5.7	18.9	1	1.3	21.2
95% CI	-	0.3-3.3	0.3-3.9	-	0.7-44.2	2.6-138.6	-	0.1-14.5	2.9-156.1
P-value	-	0.915	0.881	-	0.059	0.000	-	0.826	0.000

## Discussion

In India, the vaccination program for COVID-19 began with the vaccination of healthcare workers [12]. They were prioritized for vaccination because they carried the highest risk of COVID-19 in the process of providing care to COVID-19-positive people. Later in February 2021, frontline workers were vaccinated because they were second most vulnerable to COVID-19 infection because of their work profile [13]. The elderly and those suffering from comorbidities were identified as the most vulnerable to COVID-19 infection; therefore, in March 2021, the elderly population (>60 years) and people >45 years of age with comorbidities were vaccinated [14]. By the end of April 2021, the vaccination program picked up the pace with the arrival of the second wave (Delta) [15]. Finally, in May 2021, everyone over 18 years old also became eligible to receive COVID-19 vaccination as per Government of India directives [12,16].

In the current study, considerable variation was observed in the age profile of study participants, and this variable was not comparable among the three categories of participants. A maximum number of participants among healthcare workers (87.5%) and frontline workers (50%) were young because they were vaccinated in January, February, and March of 2021 for being at high risk. In a study conducted by Singh et al. in Bihar among vaccinated beneficiaries, it was reported that the majority of beneficiaries were healthcare workers (78%) and almost half were less than 30 years of age [17]. Another study conducted by Nguyen et al. in the US and UK reported that one-third of the beneficiaries were healthcare workers with a mean age of 42 years [18].

Vaccines, despite being protective, always carry some adverse effects [8]. In the present study, participants reported fever as the most common symptom after the first and second doses. A study conducted by Malhotra et al. in Patiala reported mild-to-moderate fever among most vaccinated healthcare workers [19]. Similarly, in a study conducted by Mittal et al. in Uttar Pradesh among adults aged more than 18 years attending health centers, it was observed that the most commonly reported symptom after the first dose of COVISHIELD was fever (30.5%) [20].

In the present study, symptoms like aches and pains were reported more often after the first dose among all the categories of participants. This finding aligned with a survey done by Jayadevan et al., who reported tiredness and myalgia as the most prevalent symptoms post-vaccination [21]. However, studies conducted by Elangovan et al. amongst healthcare workers in a tertiary care hospital in southern India reported pain at the injection site as the most common adverse effect after the first dose of vaccination followed by fever and body pain [22].

Breakthrough infections among vaccinated persons are a major concern with the emergence of variants [23]. In the present study, the incidence of breakthrough infection was significantly higher among healthcare workers compared to the general public after the second dose. However, there was no significant difference observed in the incidence of breakthrough infection after the first dose among the three categories of study participants.

Similarly, a study conducted by Singh et al. in Bihar reported that healthcare workers and frontline workers had an increased risk of getting a breakthrough COVID-19 infection after a second dose [17]. A study conducted by Ghosh et al. among healthcare workers and frontline workers of the Indian Armed Forces observed that the corrected incidence ratio of breakthrough infections was higher among unvaccinated workers than among vaccinated. However, it was also observed in this study that incidence was marginally higher among those who were fully vaccinated as compared to those who were partially vaccinated [24]. In contrast, Patil et al., in a study among vaccinated healthcare workers at a tertiary care hospital in Mumbai, reported that the percentage of vaccinated healthcare workers infected with COVID-19 was more than one-fifth after the second dose of vaccination [25]. This could be because the majority of healthcare workers were fully vaccinated by April with the arrival of the Delta wave, and their exposure to infection was at a maximum while taking care of COVID-19-positive patients. There is an increased possibility of breakthrough infection if there is increased circulation of the virus in the community even when vaccination rates are high [26]. In the present study, no deaths were reported in the two categories of healthcare workers and frontline workers on follow-up. Although vaccination cannot prevent Delta variant infection and morbidity, worldwide data have revealed that the full-course vaccinated population has been effectively protected against severe illness and death [27].

Strength of the study

This longitudinal study design has shown the incidence and relative risk of breakthrough COVID-19 infection amongst the three categories of the population. It further highlights the high vulnerability of healthcare workers, despite being vaccinated.

Limitations

Because the participants were asked via telephone about the breakthrough infection after vaccination, underreporting of breakthrough infection by the participants could be a limitation of the study because they might not have disclosed the information of positive status, given the stigma attached to it.

## Conclusions

This study revealed an increased incidence of breakthrough infection in healthcare workers as compared to frontline workers and the general community after the second dose of vaccination. Increased incidence of breakthrough infection in healthcare workers post-vaccination demands special attention, and it is important to look into various factors leading to increased risk. Healthcare workers are always vulnerable irrespective of the type of epidemic or pandemic and variant circulating in the environment. Further research is required to study the risk factors (social or behavioral) for the prevention of breakthrough infections.
